# Multivariate Longitudinal Shape Analysis of Human Lateral Ventricles during the First Twenty-Four Months of Life

**DOI:** 10.1371/journal.pone.0108306

**Published:** 2014-09-29

**Authors:** Lucile Bompard, Shun Xu, Martin Styner, Beatriz Paniagua, Mihye Ahn, Ying Yuan, Valerie Jewells, Wei Gao, Dinggang Shen, Hongtu Zhu, Weili Lin

**Affiliations:** 1 Biomedical Research Imaging Center, University of North Carolina at Chapel Hill, Chapel Hill, North Carolina, United States of America; 2 Department of Computer Science, University of North Carolina at Chapel Hill, Chapel Hill, North Carolina, United States of America; 3 Department of Psychiatry, University of North Carolina at Chapel Hill, Chapel Hill, North Carolina, United States of America; 4 Department of Biostatistics, University of North Carolina at Chapel Hill, Chapel Hill, North Carolina, United States of America; 5 Department of Biostatistics, St. Jude Children's Research Hospital, Memphis, Tennessee, United States of America; 6 Department of Radiology, University of North Carolina at Chapel Hill, Chapel Hill, North Carolina, United States of America; Institute of Psychology, Chinese Academy of Sciences, China

## Abstract

**Background:**

Little is known about the temporospatial shape characteristics of human lateral ventricles (LVs) during the first two years of life. This study aimed to delineate the morphological growth characteristics of LVs during early infancy using longitudinally acquired MR images in normal healthy infants.

**Methods:**

24 healthy infants were MR imaged starting from 2 weeks old every 3 months during the first and every 6 months during the second year. Bilateral LVs were segmented and longitudinal morphological and shape analysis were conducted using longitudinal mixed effect models.

**Results:**

A significant bilateral ventricular volume increase (p<0.0001) is observed in year one (Left: 126±51% and Right: 145±62%), followed by a significant reduction (p<0.02) during the second year of life (Left: −24±27% and Right: −20±18%) despite the continuing increase of intracranial volume. Morphological analysis reveals that the ventricular growth is spatially non-uniform, and that the most significant growth occurs during the first 6 months. The first 3 months of life exhibit a significant (p<0.01) bilateral lengthening of the anterior lateral ventricle and a significant increase of radius (p<0.01) and area (p<0.01) at the posterior portion of the ventricle. Shape analysis shows that the horns exhibit a faster growth rate than the mid-body. Finally, bilateral significant age effects (p<0.01) are observed for the growth of LVs whereas gender effects are more subtle and significant effects (p<0.01) only present at the left anterior and posterior horns. More importantly, both the age and gender effects are growth directionally dependent.

**Conclusions:**

We have demonstrated the temporospatial shape growth characteristics of human LVs during the first two years of life using a unique longitudinal MR data set. A temporally and spatially non-uniform growth pattern was reported. These normative results could provide invaluable information to discern abnormal growth patterns in patients with neurodevelopmental disorders.

## Introduction

The human brain undergoes highly dynamic growth during the first few years of life [1], reaching 80 to 90% of the adult brain size by age 2 [2], and 95% by age 6 [3]. Therefore, brain development during the first few years of life is of critical importance; abnormal growth and/or injury during this critical period can have profound implications on long-term neurological and cognitive development. Abnormal growth of the head size resulting from atypical increased gray and white matter volumes [4] during early infancy have been linked to autism [4]–[6]. In addition, abnormal enlargement of lateral ventricles has also been observed in both neurodevelopmental and psychiatric disorders including autism [7], idiopathic and syndromal mental retardation [8], fragile X syndrome [9], Down's syndrome [10], and schizophrenia [11], [12]. More importantly, it has been implicated that the abnormal enlargement of lateral ventricles could have occurred during pre- and perinatal brain development. Therefore, the ability to discern abnormal from normal lateral ventricular growth during the first years of life may offer a means to identify children at high risk for neurodevelopmental disorders. However, despite the potential clinical significance of understanding ventricular growth during early infancy, to the best of our knowledge, there have been no studies providing detailed insights into temporal and spatial development of human lateral ventricles during the first two years of life. Several early studies utilizing 2D ultrasound reported substantial volume increase during early infancy [13]–[15]. In contrast, magnetic resonance imaging (MRI) has become one of the premier tools for quantitative and noninvasive investigation of early brain development thanks to its versatility in providing detailed anatomical information without radiation exposure [16], [17]. Gilmore et al. [18], [19] reported that the left is larger than the right ventricle at birth and this asymmetry persists at 6 months of life [20]. Both Knickmey et al. [1] and Lyall et al. [21] et al reported a marked increase in ventricular volumes during the first year, followed by a reduction in the second year of life.

While the above 2D ultrasound studies and MR volumetric measures of ventricles have revealed valuable insights into the temporal growth of ventricles, these studies are limited in several different aspects. The inability to cover the entire ventricles and the difficulty to consistently examine the same anatomical locations across subjects using 2D ultrasound may have led to the inconsistent results reported in the literature regarding the temporal growth of lateral ventricles. In contrast, volumetric MR studies can only provide information pertaining to global changes. In addition, the majority of these previously reported studies (using either 2D ultrasound or MRI) have utilized a cross-sectional design and the few longitudinal studies employed a relatively long time interval between two imaging sessions (>1 yr) with respect to the temporal dynamic of brain growth. Considering the first two years of life represent the most dynamic and critical phase of human brain development and its growth during this time period has been shown to be both spatially and temporally non-uniform [22], approaches capable of discerning structural growth on a regional basis and longitudinal studies with a relatively short time interval between two contiguous imaging sessions will be needed so as to capture the temporal and spatial growth characteristics of human lateral ventricles during this critical time period. To this end, longitudinal MR images obtained from a cohort of healthy and normal infants who were imaged started from birth and every 3 months during the first year and every 6 months during the second year of life were analyzed. In addition, instead of focusing on volumetric measures, shape analysis of the lateral ventricles, which has been shown to provide more detailed structural characterization was employed in our study. Specifically, Bookstein et al. [23] constructed statistical shape models to accurately localize subtle differences in corpus-callosum shapes between schizophrenia patients and normal controls. Styner et al. [24] employed shape analysis to investigate disease and genetic effects on the shape of the lateral ventricles. Together, our studies represent, to the best of our knowledge, the first reported results providing detailed insights into temporal and spatial growth of human lateral ventricles during a critical time period of brain development with a highly unique imaging data set.

## Material and Methods

### Subjects and Imaging

A total of 24 healthy full-term infants were recruited and repeatedly imaged approximately every 3 months starting at 2 wk old during the first year of life and every 6 months in year two. To ensure only healthy subjects were enrolled in our study, the inclusion criteria were gestational age of 35 and 42 weeks, appropriate weight for gestational age, and the absence of major pregnancy and delivery complications. Additionally, the exclusion criteria were maternal pre-eclampsia, placental abruption, neonatal hypoxia, any neonatal illness requiring greater than a 1-day stay at a neonatal intensive care unit, mother with HIV, mother using illegal drugs/narcotics during pregnancy, and any chromosomal or major congenital abnormality. Informed consent was obtained from the parents of all participants and the experimental protocols were approved by the Institutional Review Board, University of North Carolina at Chapel Hill.

All subjects were scanned on a 3T MR scanner (Siemens Medical System, Erlangen, Germany) housed in the Biomedical Research Imaging Center. The T2-weighted images used in this study were acquired using a turbo spin-echo (TSE) sequence: TR  = 7380 ms, TE  = 119 ms, Flip Angle  = 150°, and resolution  = 1.25×1.25×1.95 mm^3^. A total of 70 slices were acquired to cover the entire brain. None of the subjects were sedated for MRI; all scans were performed with subjects during sleep. Subjects were fed before scanning, then swaddled, allowed to fall asleep, fitted with ear protection and their heads secured.

### Segmentation of the Lateral Ventricles

**Figure 1:**
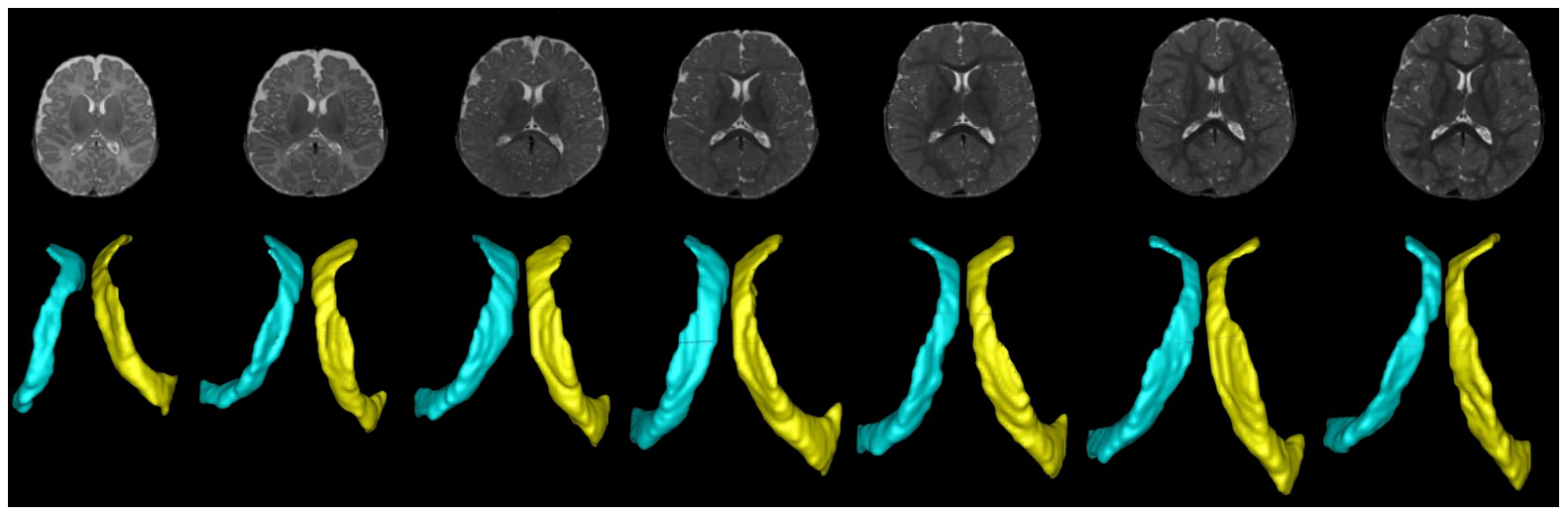
Representative T2-weighted images (upper row) from a subject imaged over the course of the first two years of life along with the segmented left and right ventricles (lower row) are shown.

T2-weighted images (Fig. 1) were used to segment lateral ventricles (LVs) thanks to the high contrast (superior to that of T1-weighted images in neonates) between cerebrospinal fluid (CSF) and brain parenchyma (white and gray matter). The images were pre-processed, including removal of non-brain tissues such as the skull and dura using Brain Surface Extractor (BSE) [25], bias correction using the non-parametric non-uniform intensity normalization (N3) method [26], and resampling to a resolution of 1×1×1 mm^3^. A longitudinal neonatal brain image segmentation algorithm [27] was subsequently applied for automatic tissue segmentation to separate brain tissues into gray matter, white matter and CSF. With the segmented CSF maps, the LV structures were carefully outlined by one of the authors. Two observers performed manual correction of the lateral ventricle segmentation using the ITK-SNAP software [28]. Since the posterior horns are not consistently present, nor is their connection to the lateral ventricle consistently identified due to the small size at this young age (Fig. 1), the posterior horns were manually removed by one of the co-authors (VJ) and excluded from subsequent data analysis. In addition, the left and right ventricles were manually separated. To determine how manual removal of the posterior horns may introduce experimental variability, 10% of the right LVs from all subjects were randomly chosen for test-retest to determine the consistency of manual removal of horns using the dice ratio [29].

### SPHARM-PDM shape correspondences

A densely sampled surface representation and surface correspondence were established using the 3D spherical harmonic based point distribution model (SPHARM-PDM) proposed by Brechbühler et al. [30] and implemented by Styner et al. [31] to compute shape descriptions and morphological information of the segmented LVs. Specifically, the segmentation maps were first processed to fill any interior holes with a minimal smoothing operation. The processed binary segmentations were converted to surface meshes, and a spherical parameterization was computed for the surface meshes using an area-preserving, distortion-minimizing spherical mapping. Using the first order ellipsoid from the spherical harmonic coefficients, the spherical parameterizations were aligned using Procrustes alignment to establish shape correspondence across all surfaces. Surface points mapping to the same positions in the spherical parameter space were considered in correspondence. The SPHARM description was then sampled into a triangulated surface (SPHARM-PDM) via an icosahedron subdivision of the spherical parameterizations. The spherical parameterization was employed to derive a medial representation of the surface called mean latitude axis [32]. The mean latitude axis was determined by linking medial points, which were computed by averaging surface points at equidistant iso-latitude parametrization. At each medial point, the local average radius and average cross-sectional area were measured. The Euclidean distances between neighboring medial axis points represent local length of the LVs. All of these parameters were used to characterize how the shapes of the LVs are altered with age.

### SPHARM-PDM shape correspondences validation

Since the main goal of our study was to assess the temporal and spatial LV shape characteristics during the first two years of life through a longitudinal study, it is imperative to determine the accuracy of the SPHARM-PDM established correspondence of the same subjects but at different ages. A landmark approach was implemented to compare surface correspondences established by SPHARM-PDM. Three subjects were randomly selected from the dataset; each had 5 time points (2 weeks to 12 months) for a total of 15 shapes. We randomized these datasets, used the Slicer 3D tool to visualize the surfaces, and manually located 10 predefined locations, including 3 at the corner and turning points on each of the two horns and 4 at the mid-body (2 points on each side around the 1/3 and 2/3 of the LV body). The surface of subject 3 at 6 months old was chosen as the template to locate the corresponding points as determined by SPHARM-PDM correspondence on the remaining surfaces. Finally, we computed the differences between SPHARM-PDM and the manually defined correspondences.

### Longitudinal Analysis

We applied the linear longitudinal mixed models proposed by Laird and Ware [33] to conduct volumetric, morphological, and shape statistical analysis using R 3.0.0 software with the lmer package [34].

For the volumetric analysis, we first considered Age and Age^2^ as fixed effects and a random intercept for each of left and right volumes. Before fitting the models, the age and volume were normalized. To compare left and right volumes, we considered both volumes in the same model, simultaneously. For the *i^th^* subject at age *j*, a linear mixed model was considered as the following:

For Left LV: 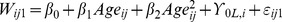



For Right LV: 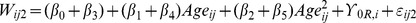



The 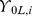 and 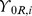 were random intercepts for the left and right LVs, respectively. The variances of the left and right volumes were allowed to be different, enabling statistically testing the significance of *β_3_*, *β_4_* and *β_5_* and investigating the difference between the left and right volumes.

For the morphological analysis, a functional mixed effect modeling framework (FEME) [35] was used to jointly analyze the repeatedly measured length, radius and area along the medial axis of LVs and the covariates of interest. We considered a model with a fixed piece-wise linear growth curve of age and a random intercept. The coefficients and covariance were modeled as functions of location along the LVs to reflect spatially different effects of covariates with age as the covariates. Regional and global tests were performed for length, radius, and area, separately, to test both point-wise as well as overall effects of age.

As for the longitudinal shape statistics, we considered Gender, Age, Age^2^, and Gender*Age as fixed effects and random intercepts. We hypothesized that the LV shape may change in different directions with age. Thus, the intercepts and slopes may be different according to the direction. In addition, we also assumed that each subject had different random intercepts for each of three directions (left-right, anterior-posterior, and superior-inferior corresponding to x, y, and z directions hereafter); that is, each subject was allowed to have their own variances depending on (x, y, z) directions. Let *W_ijk_* represents x, y, or z coordinate for k = 1, 2, or 3, respectively. The subscripts i and j indicate the *i^th^* subject at age *j*. A multivariate linear mixed model was considered as the following:
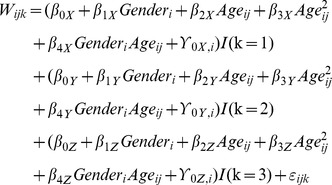



Before fitting the models, ‘age’ was normalized and ‘W’ was centered. Furthermore, ‘age in days’ instead of in months was used where ‘age’ was included in three variables (age, age∧2, age*gender) so as to examine the pure effects of age and gender while controlling other effects. Additionally, to test bilateral shape differences, we calculated the difference between left and right LVs where the right LV was flipped to the left beforehand.

In order to reveal the developmental growth patterns of LVs, the major growth direction of LV was obtained point-by-point. Specifically, the major growth direction at each corresponding point was computed by performing singular value decomposition (SVD) of the individual difference vectors between the later time points and the first time point at each correspondence. These individual difference vectors contain information of local growth trend, since SVD provides the optimal average local growth direction. Subsequently, the growth rate along each direction can be obtained to examine the developmental pattern.

Corrections for multiple comparison were accomplished whenever needed using False Discovery Rate [36] for all of the above outlined statistical analyses.

## Results

**Figure 2:**
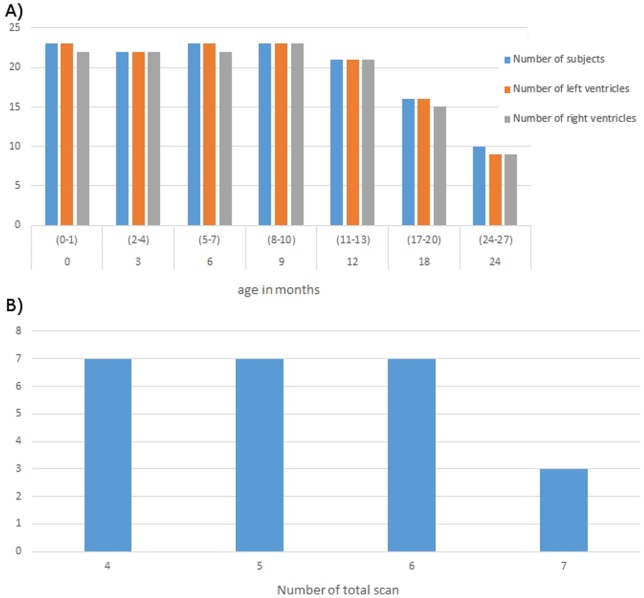
The number of subjects imaged and the number of right and left ventricles available for analysis at each age point is shown in Fig. 2A. The labels of x-axis in (A) indicate the age range when the subjects were scanned. In contrast, B shows the number of subjects who underwent different numbers of imaging sessions. The labels of x-axis in (B) indicate the number of time that subjects were imaged.

Fig. 2a provides information regarding the number of subjects at each age as well as the number of segmented left and right LVs, respectively. In contrast, Fig. 2b provides the number of subjects for different numbers of scans. Note that there are missing time points from our study cohort. The reasons for missing data include missing appointments (n = 2 at 3 and 12 months; n = 1 at 6, 9, and 18 months), subject attrition (n = 4 at 24 months), motion (n = 1 at 2 weeks), subjects had not reached the next target age at the time of the study (n = 1 at 12 months; n = 7 at 18 months; n = 10 at 24 months) and failure of SPHARM-PDM (n = 1 at 2 weeks, 6 months, and 18 months; n = 2 at 24 months). Therefore, of 168 pairs of LVs from 24 subjects, a total of 137 left and 133 right ventricles were included in the study. Since these underlying reasons for missing data are independent of the variables of interest, they should not affect the conclusions of this study.

### Shape segmentation validation

As mentioned above, leave-10%-out cross-validation was employed to determine the potential variability resulting from manual removal of the tails of ventricular horns. The average dice ratio is 0.99±0.04, suggesting highly consistent removal of the tails of the ventricular horns. A representative example overlaying the original (red) and the validation (blue) ventricles of a subject is shown in Fig. S1.

### SPHARM-PDM correspondence validation

**Figure 3:**
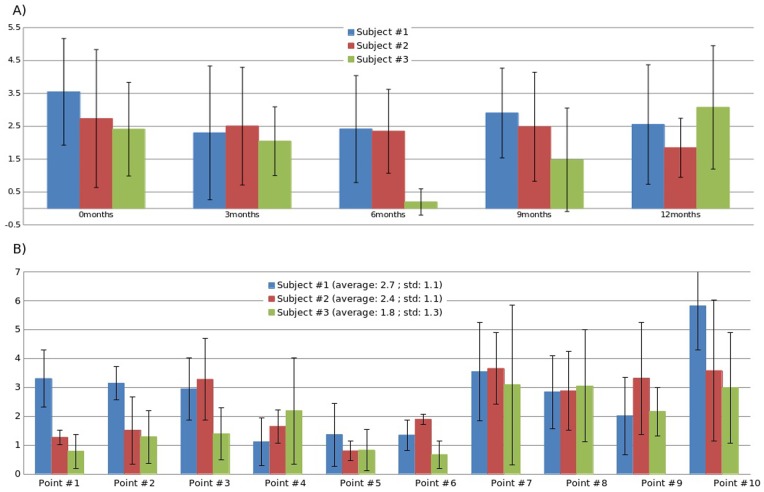
Distance differences in millimeters (from center to center) of the 10 predefined landmarks between manually and SPHARM-defined correspondences with age for each subject (A). The error bars in A represent the standard deviations of the distance differences across the 10 predefined landmarks. In contrast, B shows the distance differences of each landmarks for the three subjects, respectively. The error bars here represent the standard deviations across age. The points 1–6 in x-axis were located in the horn while the points 7–10 were located in the body of LVs.

Figure 3 reports the distance differences between manually and SPHARM-defined correspondences among three randomly selected subjects, respectively. Fig. 3A shows the distance differences with age among the 10 predefined landmarks for each subject. In contrast, Fig. 3B shows the distance differences of each landmark among the five time points. It is apparent that the average distance differences between the two methods are relatively stable across all ages with the exception that subject 3 shows the smallest differences at 6 months old (Fig. 3a). Furthermore, the distance differences located at the mid-body (points 7–10) are larger (3.2±1.8 mm) than that at the horns (1.7±1.2 mm) (Fig. 3b). Overall, the average distance differences of all subjects is 2.3±1.6 mm, suggesting that the SPHARM-PDM approach is capable of achieving accurate correspondence of longitudinal analysis. Finally, the standard deviations reported here imply that although the distance differences between manually and SPHARM-defined correspondences are relatively small, there are some variability among landmarks and with age.

### Volumetric Analysis

**Figure 4:**
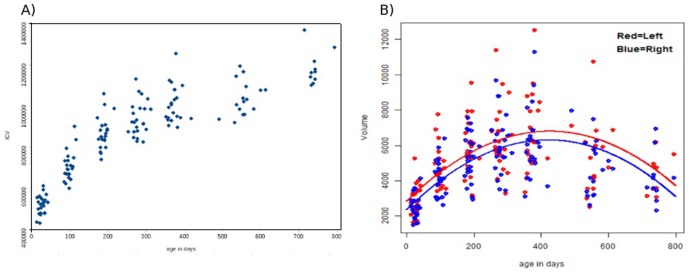
The total intracranial volume (ICV) and the left and right ventricular volumes with age are shown in A and B, respectively. The red and blue filled circles in B represent the left and right ventricles, respectively.

The intracranial (Fig. 4a) and ventricular (Fig. 4b) volumes during the first two years of life are shown in Fig. 4. Consistent with the previously reported results, ICV (the sum of CSF, gray matter and white matter volumes) shows a marked increase in year 1, followed by a slower increase in year 2. In contrast, the growth of the lateral ventricles exhibits a biphasic behavior with a significant increase during the first year of life (Left: 126±51% (p<0.0001) and Right: 145±62% (p<0.0001) from two weeks to 1 yr of age), following by a significant reduction from 1 yr to 2 yr of age (Left: −24±27% (p = 0.02) and Right: −20±18% (p<0.01)). In addition, the most dramatic growth appears to be the first three months of life (Left: 52.06±24.65% (p<0.0001) and Right: 56.25±24.53% (p<0.0001), followed by 3–6 months(Left: 28.52±16.03% (p<0.0001) and Right: 33.29±16.62% (p<0.0001)), and 6–9 months(Left: 12.40±12.78% (p = 0.0002) and Right: 14.52±20.46% (p = 0.0032)). After 9 months of age, the volumetric changes (increase or decrease) of the lateral ventricles between two adjacent imaging time points become more subtle (p>0.05). Finally, although the growth trajectories appear to be different between the left and right ventricles starting from birth, with the left ventricle exhibiting a larger volume than that of the right, the longitudinal mixed models show that none of the parameters (intercept, age, and age^2^) are significant (p>0.05).

### Morphological Analysis

**Figure 5:**
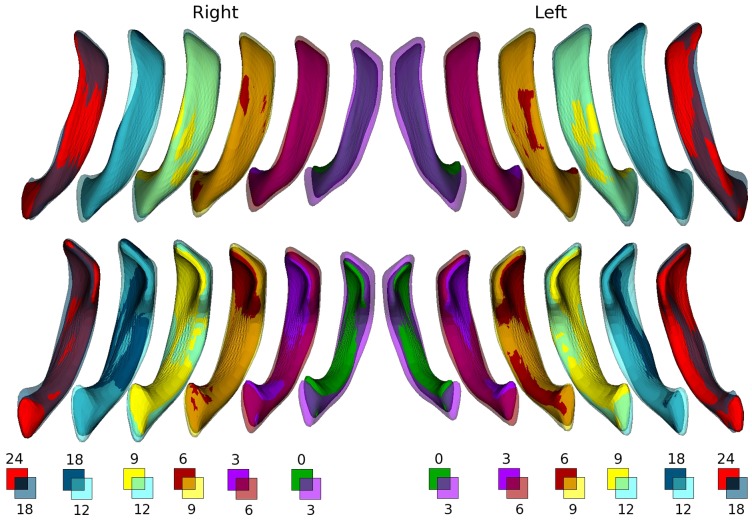
Qualitative comparisons of the shape changes of the right and left lateral ventricles between two contiguous imaging time points are shown. The correspondence between the color scheme and the age is provided at the bottom of the figure. The opacity of the color is adjusted based on the size of the ventricle such that a high opacity is used for the larger ventricle of the two time points whereas the smaller ventricle is shown using a solid color. Since the volumes of both the left and right ventricles increase in year 1, solid color is used for the older time point and a color with a high opacity is used for the younger time point whereas this scheme is reversed for the comparisons in year 2 where the LV volumes are reduced with age.

While volumetric measurements provide quantitative assessments of the growth trajectories of LVs, it lacks spatial information regarding the underlying morphological changes contributing to the observed volumetric changes. Fig. 5 provides qualitative comparisons of morphological alterations of LVs between two adjacent time points for the left and right ventricles, respectively. Several important growth features are observed. The major growth of the LVs appears along the anterior-posterior direction (lengthening) in the first year of life. In addition, the LVs become thicker along the dorsal-ventral direction with age. Finally, the sizes of the LVs appear to be uniformly reduced between 12–18 months, followed by a more regionally specific reduction in size between 18–24 months.

**Figure 6:**
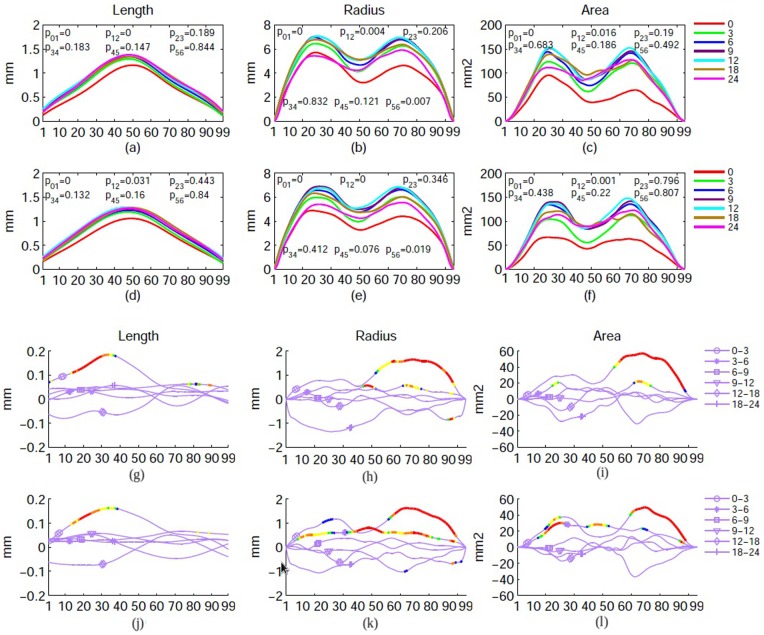
Mean length, radius, and area of the left (a–c and g–i) and right (d–f and j–l) LVs are shown. The x-axis indicates the anatomical location along the long axis of LV, with point 1 representing the anterior and point 99 the posterior tips. Fig. 6a–f show the raw morphological parameters of the entire LV. The P_ij_ is the p-value comparing between two contiguous time points for a given morphological parameter, where i and j represent the imaging time points and i≠j. Fig. 6g–l provide the differences of mean length, radius, and area between two adjacent time points. Colors indicate different p-values with red (<0.01), orange (0.01<p-value<0.02), yellow (0.02<p-value <0.03), green (0.03<p-value<0.04), blue (0.04<p-value<0.05), and light purple (p-value>0.05), respectively.

Quantitative analyses of longitudinal LV morphological changes (length, area, and radius) with age are shown in Fig. 6, illustrating that the growth patterns of LVs are spatially inhomogeneous and not congruent. A significant bilateral growth during the first 6 months of life was apparent for three morphological parameters of the entire LVs (Fig. 6a–c, left LV and Fig. 6d–f, right LV). Moreover, a significant bilateral increase of radius from 18 to 24 months is also observed (Fig. 6b and 6e). To further determine spatial heterogeneities of morphological growth patterns, the difference of each morphological parameter between two adjacent time points was obtained (Fig. 6g–l) and a point-wise comparison was conducted. Additional insights into spatially variant growth patterns are observed through the piecewise comparisons. Significant lengthening of the anterior LVs (points 10–40) was observed bilaterally during the first 3 months. In contrast, the increase of radius and area appears largely in the posterior LVs (points 50–99) from 0–3 months, followed by a more spatially uniform increase from 3–6 months.

### Shape Characteristics

**Figure 7:**
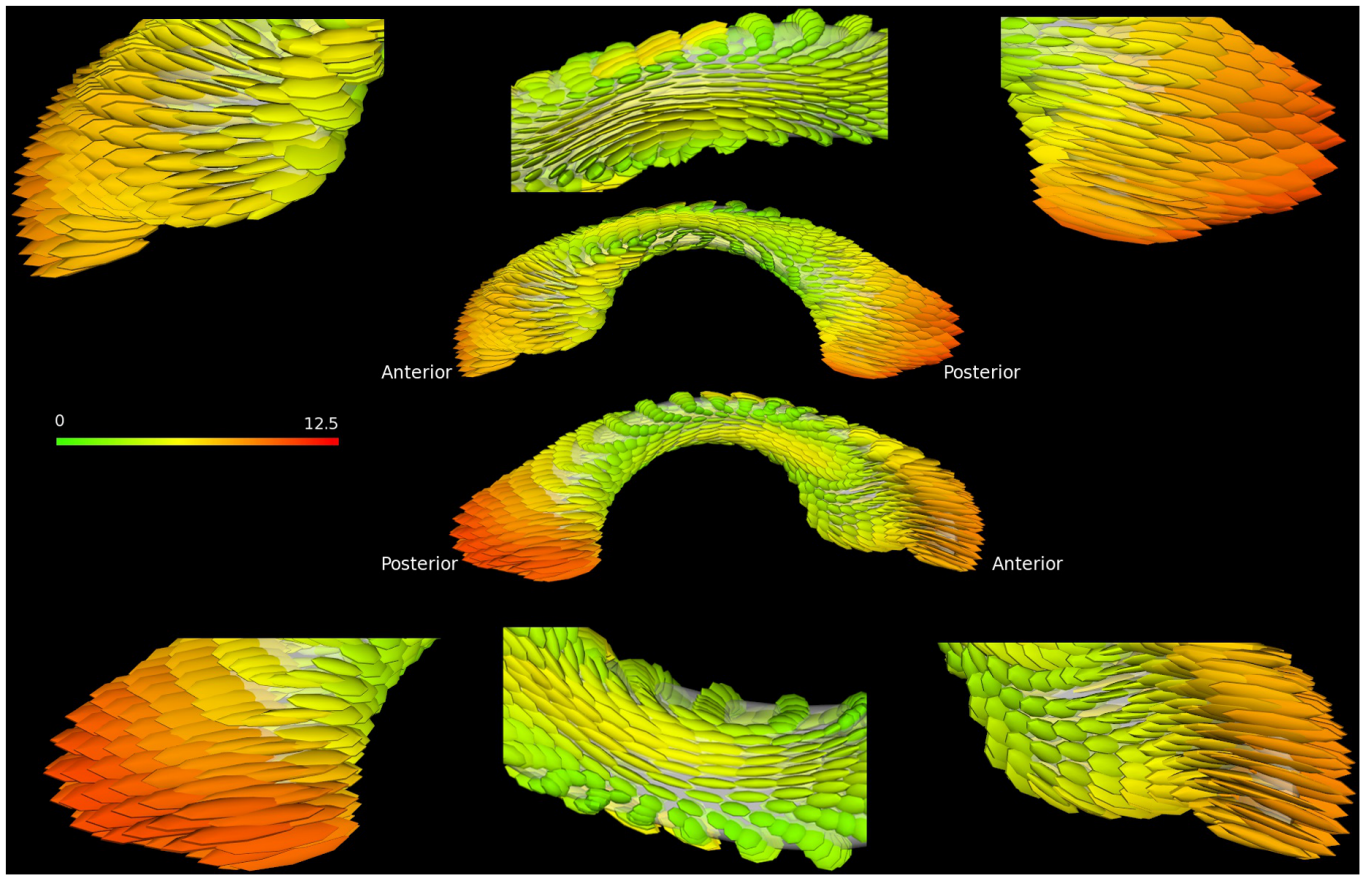
The major growth direction and growth rate of the left LV are shown. The long axis of the ellipsoids shows the main growth direction, while the length and color demonstrate the growth rate from the first and last imaging time points. The color-bar represents the magnitude of the growth rate.

The major growth directions (long-axis of the ellipsoid) as well as the growth rates (color and the length of the ellipsoids) of the left LVs are shown in Fig. 7 for the dorsal (upper panel) and ventral (lower panel) surfaces. (The right LV shows a similar characteristic as that observed in the left LV and thus is not shown here.) Three important features emerge. The major growth direction of the body of LVs is along the anterior-posterior direction with a similar growth rate for both dorsal and ventral surfaces. In contrast, the growth direction of the horns is norm to its surface. Finally, the growth rates are faster in the horns than in the mid-section of the LVs.

**Figure 8:**
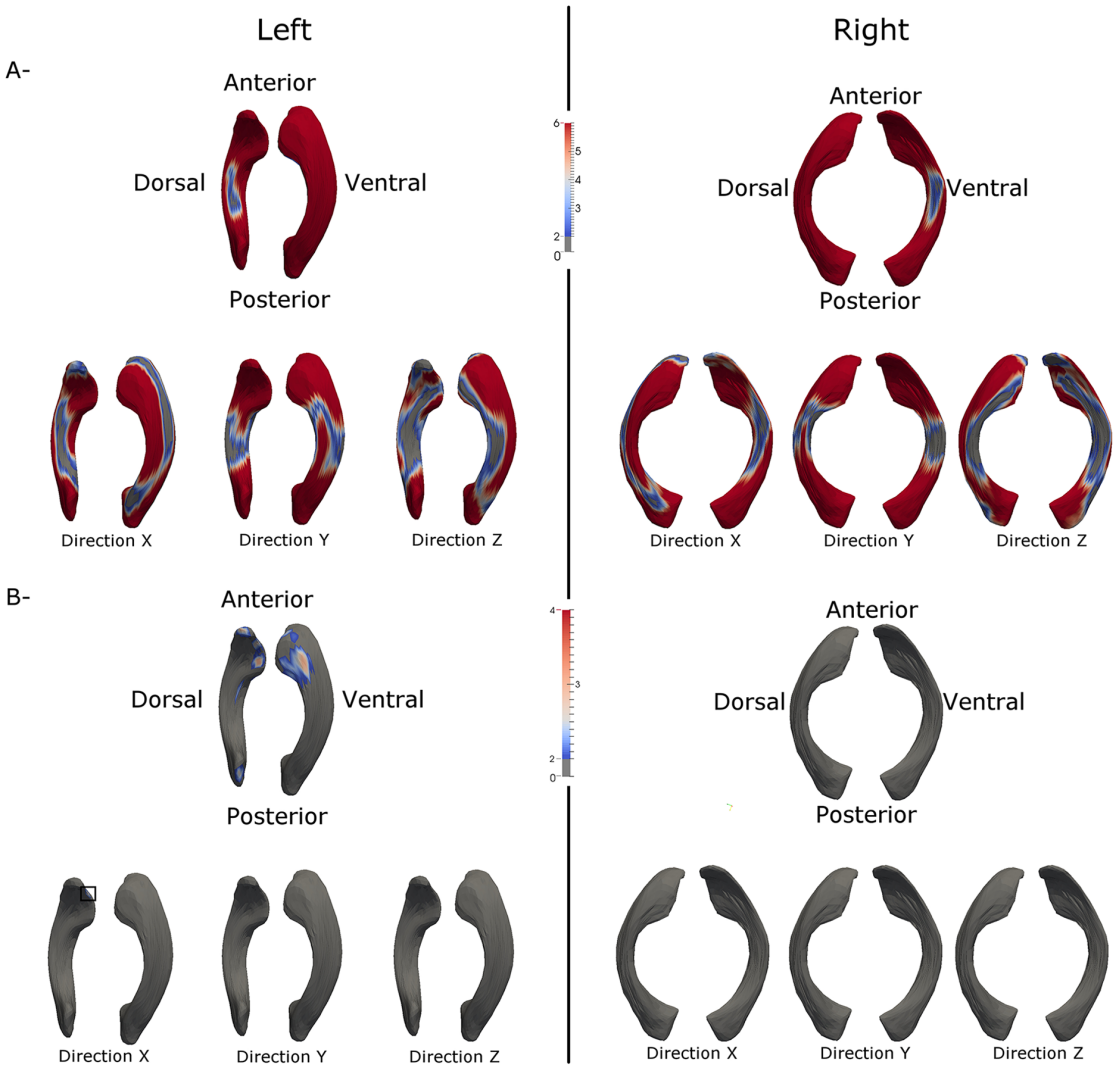
The effects of age and gender on shape growth of the ventricles. (A) The effects of age on the shape growth of the left and right ventricles are shown, respectively. The upper row shows the effects without considering the direction of growth while the age effects on the growth of ventricle along the left-right (X), anterior-posterior (Y), and superior-inferior (Z) directions are shown in the bottom row, respectively. The (–log10(p-value)) corrected for multiple comparison using FDR is overlaid on the ventricles and the color bar represents the –log10(p-value). (B) The effects of gender on the shape growth of the left and right ventricles are shown, respectively. The organization of Fig. 8B is identical to that shown in A.

Age (Fig. 8A) and gender (Fig. 8B) effects on the shape growth of LVs are shown in Fig. 8, respectively. Without considering the growth direction of the ventricle, significant bilateral age effects are observed (p<0.01) for the entire ventricles with the exception of the small regions located at the mid body of the ventricles (upper row, Fig. 8A). More importantly, the age effects are growth directionally dependent with significant effects at both the anterior and posterior horns along the anterior-posterior direction (Y-direction) whereas significant age effects along both the X and Z directions are present throughout the entire ventricles. In contrast, gender effects are more subtle and significant effects only present at the left anterior and posterior horns (upper row, Fig. 8B) when the growth direction is not considered. Furthermore, significant gender effects are only observed at a small region located on the left anterior horn along the x-direction (square box).

**Figure 9:**
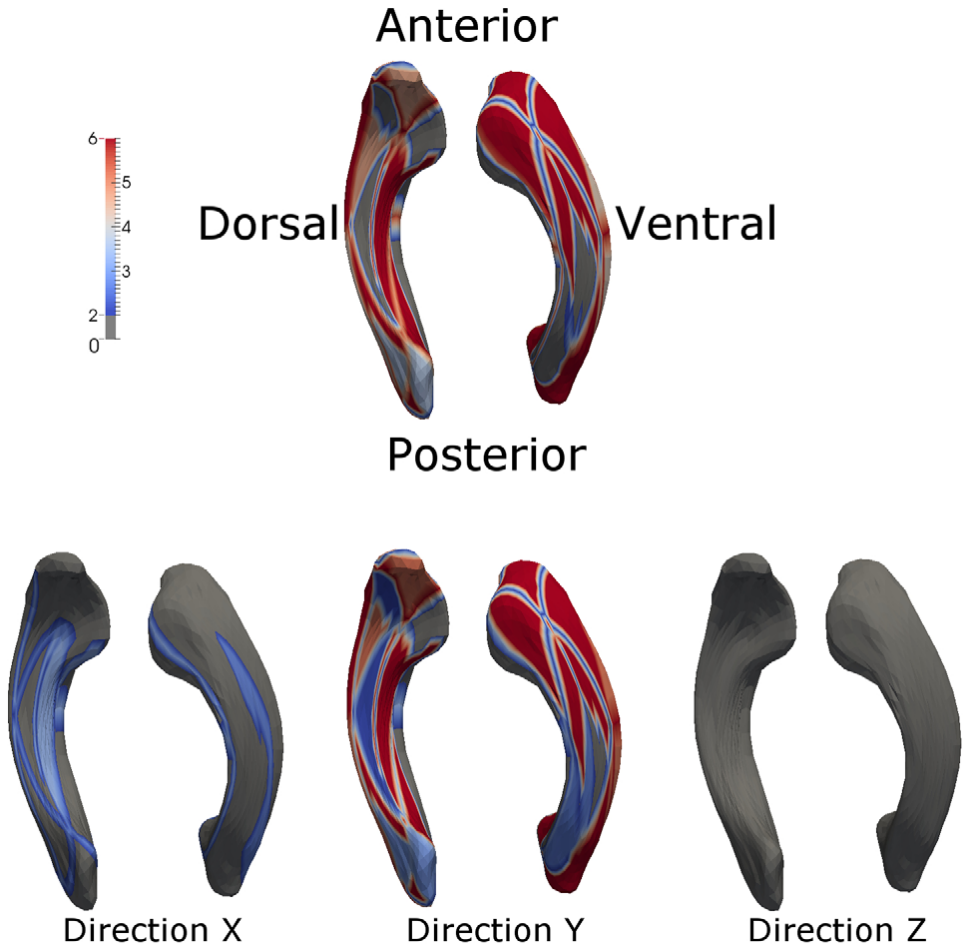
The effects of age on the shape differences between the left and right ventricles with age are shown. The upper row shows the effects without considering the direction of growth while the age effects on the growth of ventricle along the left-right (X), anterior-posterior (Y), and superior-inferior (Z) directions are shown in the bottom row, respectively. The –log10(p-value) corrected for multiple comparison using FDR is overlaid on the ventricles and the color bar represents the –log10(p-value).

Finally, the age effects on the shape differences between the left and right LVs are shown in Fig. 9. Interestingly, although significant bilateral age effects are observed throughout the entire ventricles without considering the growth direction, the main effects appear to be along the Y direction, followed by X direction whereas no significant effects are observed along the Z-direction.

## Discussion

While previous studies focusing on volumetric measurements of LVs have offered some understanding about the growth patterns of the LVs, volumetric assessments only provide global information. In addition, since our brain undergoes perhaps the most significant growth during the first years of life [1], a temporally sparse imaging acquisition approach may not provide detailed temporal and spatial characteristics of LV growth during this critical time period of brain development. To this end, we reported the temporal and spatial shape characteristics of the lateral ventricles during the first two years of human life through a longitudinal MR study of healthy and normal infants. The subjects were imaged starting at birth, every three months during the first year of life, and every 6 months during the second year. Shape characteristics of the LVs were evaluated through geometric boundary-based dense representation. Multivariate longitudinal statistical models were employed to discern age, gender (Fig. 8) as well as lateralization effects (Fig. 9) on the growth of the LVs. Together, to the best of our knowledge, our study represents the first comprehensive characterization of temporal and spatial growth of human lateral ventricles during the first two years of life.

The analysis of morphological changes and local growth directions reveal important features of LV growth during the first two years of life. Specifically, the fastest growth rate occurs during the first 3 months (Left: 52.06±24.65% (p<0.0001) and Right: 56.25±24.53% (p<0.0001), followed by 3–6 months(Left: 28.52±16.03% (p<0.0001) and Right: 33.29±16.62% (p<0.0001)), 6–9 months(Left: 12.40±12.78% (p = 0.0002) and Right: 14.52±20.46% (p = 0.0032)), and becomes more stable with age (p>0.05) after 9 months of age. More importantly, a reduction in LV volume is clearly visible during the second year of life. This first increase in year 1 followed by a reduction of volume in year 2 has been previously reported. Lyall et al. [21] found a 102% increase of volume during the first year (25 subjects) of life, followed by a 7% decrease in the second year (18 subjects). Knickmeyer et al. [1] showed a similar pattern of growth but with a different growth rate; the LV size increased by 280% in year 1 and −8% in year 2. While the volumetric analyses of LVs in our study also show a similar increase in year 1 followed by a decrease of LV volumes in year 2, our results offer additional insights that are not available with volumetric assessments. Specifically, morphological analysis reveals that temporal changes of LV volumes are not spatially uniform. As shown in Fig. 5, although an overall increase in volume of LV is observed during the first 3 months of life, the anterior horn appears to enlarge more than that of the posterior horn and more along the dorsal than that in the ventral direction in year 1. These findings are supported by the statistical analysis shown in Fig. 6; a significant lengthening of the anterior LVs is observed whereas the posterior LVs exhibit a significant increase of cross-section area during the first three months of life. The estimated growth rates (Fig. 7) show that the frontal and caudal ends extend most rapidly towards the anterior and posterior directions, respectively, and the mid-body remains relatively constant over time. Interestingly, these findings appear to be consistent with the growth of the shape of the human brain. Xu et al. [22] showed that the shape of the human brain grows fastest along the anterior-posterior direction during early brain development. In addition, the maturation of white matter during early infancy has also been reported following the anterior-posterior direction [33], [37], [38].

Asymmetrical growth between the two hemispheres of human brain has been well-documented and can be detected as early as in utero [39]. While volumetric measures of LVs consistently demonstrate that left LV is larger than the right LV starting from birth and persisting throughout the first two years of life (Fig. 4), these differences are not statistically significant. Reiss et al [40] imaged 85 children from 5 to 17 using MRI and showed that the left is significantly larger than the right. Similar findings were also reported by other groups [41], [42]. While the differences in experimental design between the previous and our studies may account for the observed discrepancies, there are two plausible explanations. First, our studies focused on early brain development while toddlers or adults were studied previously. Therefore, it is conceivable that the left-right differences will become more prominent with age. Second, the modest sample size in our study may limit the statistical power to discern the volumetric differences between left and right. Along this line, the fact that volumetric assessment only provides global measures may further reduce the statistical power. Interestingly and importantly, our multivariate shape analysis reveals a significant age effect on the left-right differences (Fig. 9), demonstrating the improved statistical power using the shape analysis approach.

### Shape analysis

The most fundamental element of statistical shape analysis is the representation of shape, or shape descriptor, and its implied shape correspondences over the whole population. Various shape descriptors have been used in the literature, including parametric descriptors where a shape is expressed as coefficients of the harmonic basis functions used to parameterize the shape surface [30], [43], surface-based landmarks where each shape is configured as a finite-dimensional vector of boundary points in Euclidean space [44], medial axes where a shape is represented by its own skeleton [45]–[47], and deformation fields where an explicit geometric boundary is not present, rather, a shape is considered as the non-rigid deformation of a template to an image where the shape lays [48]. In this study, spherical harmonic based point distribution model (SPHARM-PDM) was employed where the shape correspondences were established after parameterizing each shape instance into a parameter space of unit sphere using spherical harmonic basis functions. After aligning all the shapes by their first order ellipsoid, the surface points that map to the same position on the parameter space are considered to be correspondences [30],[31]. Despite the fact that shape correspondence is a major factor in determining the accuracy of the subsequent statistical shape analysis, evaluation and comparison of different correspondences is not trivial [49]–[51], if not impossible, since all automatic shape-correspondence algorithms are developed by optimizing some assumed mathematical or physical models of their own and there is no ground-truth to compare with. Indeed, different shape-correspondence algorithms all have their own strengths and weaknesses and may be suitable for different applications depending on the scenario and the characteristics of the structure of interest. Through a landmark-based approach, we have evaluated the accuracy of the SPHARM-PDM established correspondence of the same subjects but at different ages. An average distance of 2.3±1.6 mm (the worst case around 5 mm) between manual and automatic correspondences was obtained, suggesting that SPHARM-PDM establishes accurate correspondences, allowing longitudinal assessments of shape changes associated with human LVs.

## Conclusions

Through a unique longitudinal MR data of health infants imaged starting from birth every three months during the first year of life and every 6 months in the second year of life, the temporal and spatial growth characteristics of lateral ventricle morphology and shapes were revealed. Results obtained from this study offer invaluable insights into normal growth of human lateral ventricles not only volumetric measurements but also shape statistics. These normative information is likely to have profound implications for the study of abnormal growth of LVs in neurodevelopmental and psychiatric disorders.

## Supporting Information

Figure S1 Since the posterior horns are not consistently present, nor is their connection to the lateral ventricle consistently identified at this young age (Fig. 1) owing to partial volume effects, the posterior horns were manually excluded from subsequent data analysis. Although anatomical landmarks were employed to minimize experimental confounds resulting from manual removal of the posterior horns, shape segmentation validation was conducted to determine if the manual removal of the posterior horns leads to experimental confounds. With the leave-10%-out cross-validation, a high (0.99) DICE ratio was obtained, suggesting that manual removal of the posterior horns was consistently achieved and should not contribute to experimental confounds in the subsequent shape analysis. Fig. S1 shows a representative example overlaying the original (red) and the validation (blue) segmentations of a subject. It is perhaps not surprising that only several small regions show in red or blue color and the remaining ventricle is all in purple color.
(JPG)
