# Supplementary figures and images for: Multivariate Longitudinal Shape Analysis of Human Lateral Ventricles during the First Twenty-Four Months of Life
Source: PLoS One. 2014 Sep 29;9(9):e108306. doi: 10.1371/journal.pone.0108306 (PMC4180454; doi:10.1371/journal.pone.0108306)

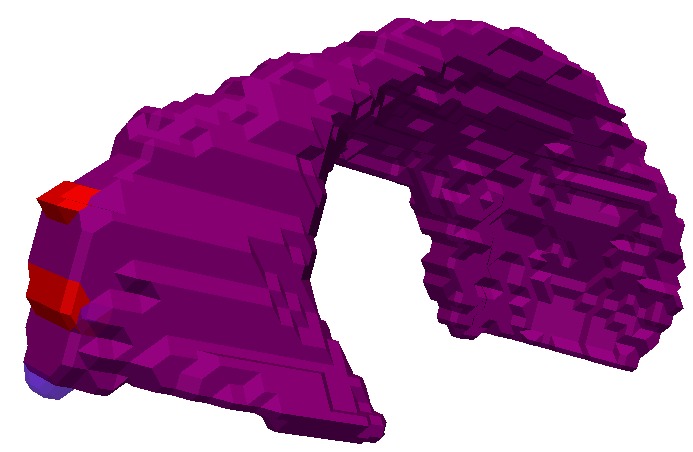

Supplement: Figure S1 — Since the posterior horns are not consistently present, nor is their connection to the lateral ventricle consistently identified at this young age (Fig. 1) owing to partial volume effects, the posterior horns were manually excluded from subsequent data analysis. Although anatomical landmarks were employed to minimize experimental confounds resulting from manual removal of the posterior horns, shape segmentation validation was conducted to determine if the manual removal of the posterior horns leads to experimental confounds. With the leave-10%-out cross-validation, a high (0.99) DICE ratio was obtained, suggesting that manual removal of the posterior horns was consistently achieved and should not contribute to experimental confounds in the subsequent shape analysis. Fig. S1 shows a representative example overlaying the original (red) and the validation (blue) segmentations of a subject. It is perhaps not surprising that only several small regions show in red or blue color and the remaining ventricle is all in purple color. (JPG) [file pone.0108306.s001.jpg]
